# Supplementary material for: vScreenML v2.0: Improved Machine Learning Classification for Reducing False Positives in Structure-Based Virtual Screening
Source: Int J Mol Sci. 2024 Nov 18;25(22):12350. doi: 10.3390/ijms252212350 (PMC11595162; doi:10.3390/ijms252212350)
Supplement: Supplementary file 1 [file ijms-25-12350-s001.zip › ijms-3263701-supplementary.pdf]

Supporting Information

vScreenML v2.0: Improved Machine Learning Classification for Reducing False Positives in Structure-Based Virtual Screening

Grigorii V. Andrianov [1,2], Emeline Haroldsen [1] and John Karanicolas [1,3,*]

[1] Cancer Signaling & Microenvironment Program, Fox Chase Cancer Center, Philadelphia, PA 19111, USA; grigorii.andrianov@gmail.com (G.V.A.); eharold1@jh.edu (E.H.)
[2] Institute of Fundamental Medicine and Biology, Kazan Federal University, Kazan 420008, Russia
[3] Moulder Center for Drug Discovery Research, Temple University School of Pharmacy, Philadelphia, PA 19140, USA
* Correspondence: john.karanicolas@abbvie.edu Tel.: +1-215-728-7067

**Filter name**	**Description**	**Number of filtered actives (applied sequentially)**
	Physicochemical properties	
Molecular weight (MW)	300 < MW < 500	31,036
Heavy atoms (HA)	HA > 19	29,219
LogP	0 < LogP < 5	14,496
	2D structural properties	
Smallest ring size (SRS)	0 < SRS <= 6	12,153
Fused rings (FR)	FR < 4	11,556
Unbranched chain (UC)	Absence of linear unbranched ` [D2&!R]~[D2&!R]~[D2&!R]~[D1,D2&!R]` substructure in ligand	9,837
Sugar	Absence of ` [#6&R1]-[#8&R]-[#6&R1]-[$([#6&R]~[#8&H&!R])]-[$([#6&R]~[#8&H&!R])]` substructure in ligand	9,725
ATP-like	Absence of nucleotide, sugar and phosphorus substructures in ligand	9,465
N/O composition	At least one nitrogen and oxygen presented in ligand	8,407
Undesired atoms	Only C, N, O, S, F, Cl or Br atoms presented atoms	7,879
Halogens	Number of ` [F,Cl,Br]` substructure < 4	7,666
	3D structural properties (applied only to actives set)	
Structural completeness (SC)	SC = 1.0	7,427
Ranking model fit (RMF)	RMF > 0.35	6,077
Ranking model geometry (RMG)	RMG > 0.2	3,484
Ligand proximity to cysteine ($d_{closest\ cys}$)	$d_{closest\ cys}$ > 1.9 A	3,387

Ligand proximity to other ligands ($d_{closest\ ligand}$)	$d_{closest\ ligand}$ > 12.0 A	1,411
Presence of nucleic acids (NA)	NA = False	1,407

Table S1. Filtering rules applied to molecules obtained from RCSB and DUD-E.

**PyRosetta**		
TotalExposedSasa	FaAtrInteraction	HBondScInteraction
TotalBSA	FaRepInteraction	GenBonded
InterfaceHydrophobicSasa	FaSolInteraction	HBInterface
InterfacePolarSasa	FaElecInteraction	InterfaceUnsat
InteractionScore	HBondBbScInteraction	
**PyRosetta and RDKit**		
NH1	NH3	carbonyl O
NH2	OH	carboxylate O
**BINANA**		
SideFlexAlpha	PiPi	TotalHphobics
SideFlexBeta	TStacking	
SideFlexOther	CationPi	
BackFlexAlpha	SaltBridge	
BackFlexBeta	TotalElec	
BackFlexOther	TotalHBond	
**RF-score**		
6.6	9.6	17.6
6.7	9.7	17.7
6.8	9.8	17.8
6.16	9.16	17.16
7.6	15.6	35.6
7.7	15.7	35.7
7.8	15.8	35.8
7.16	15.16	35.16
8.6	16.6	
8.7	16.7	
8.8	16.8	
8.16	16.16	

**RDKit**		
exactmw	NumHeterocycles	chi1n
amw	NumAromaticHeterocycles	chi2n
lipinskiHBA	NumSaturatedHeterocycles	chi3n
lipinskiHBD	NumAliphaticHeterocycles	chi4n
NumRotatableBonds	NumSpiroAtoms	hallKierAlpha
NumHBD	NumBridgeheadAtoms	kappa1
NumHBA	labuteASA	kappa2
NumHeavyAtoms	tpsa	kappa3
NumAtoms	CrippenClogP	Phi
NumHeteroatoms	CrippenMR	
NumAmideBonds	chi0v	
FractionCSP3	chi1v	
NumRings	chi2v	
NumAromaticRings	chi3v	
NumAliphaticRings	chi4v	
NumSaturatedRings	chi0n	
**LUNA**		
Proximal	Chalcogen bond	Face-to-face pi-stacking
Hydrogen bond	Chalcogen-pi	Face-to-edge pi-stacking
Ionic	Halogen-pi	Face-to-slope pi-stacking
Salt bridge	Orthogonal multipolar	Edge-to-edge pi-stacking
Cation-pi	Parallel multipolar	Edge-to-face pi-stacking
Hydrophobic	Antiparallel multipolar	Edge-to-slope pi-stacking
Halogen bond	Tilted multipolar	Displaced face-to-face pi-stacking
Repulsive	Multipolar	Displaced face-to-edge pi-stacking
Water-bridged hydrogen bond	Cation-nucleophile	Displaced face-to-slope pi-stacking
Amide-aromatic stacking	Anion-electrophile	
Weak hydrogen bond	Unfavorable anion-nucleophile	
Covalent bond	Unfavorable cation-electrophile	
Atom overlap	Unfavorable nucleophile-nucleophile	
Van der Waals clash	Unfavorable electrophile-electrophile	
Van der Waals	Pi-stacking	
**PocketDruggability**		
C_RESIDUE	hydrophobic_kyte	p_aliphatic_residue

INERTIA_3	hydrophobicity_pocket	p_aromatic_residue
SMALLEST_SIZE	p_Ccoo	p_negative_residue
SURFACE_HULL	p_N_atom	
VOLUME_HULL	p_Ooh	

Table S2. All features calculated for new version of vScreenML. Green indicates the most important features for inference.

Target	KarmaDock			RTMScore			CNNScore		
	Score	Seq in training	PDB in training	Score	Seq in training	PDB in training	Score	Seq in training	PDB in training
11betahsd1	2.51	-	-	0	-	-	5.03	-	-
17betahsd1	0	+	-	0	+	-	0	+	-
a2a	10.23	+	+	7.67	+	+	2.56	-	-
ace	15.44	+	+	2.57	+	+	12.86	+	-
ace2	0	-	-	0	-	-	0	-	-
ache	10.17	+	+	7.63	+	+	5.09	+	-
adam17	12.81	+	+	10.25	+	+	17.94	+	+
adrb2	9.59	+	-	14.39	+	-	2.4	-	-
akt1	15.15	+	+	17.68	+	+	12.63	-	-
alr2	0	-	-	0	-	-	2.55	-	-
ar	15.05	+	+	18.81	+	+	3.76	+	+
aurka	25.85	+	+	23.27	+	+	7.76	+	-
aurkb	27.84	+	+	27.84	+	+	10.12	+	-
bcl2	10.19	+	+	2.55	+	+	7.64	+	-
braf	18.08	+	+	15.5	+	+	7.75	-	-
catl	5.14	+	+	10.28	+	+	2.57	+	-
cdk2	14.95	+	-	14.95	+	-	7.48	+	-
cox1	2.61	-	-	0	-	-	5.22	-	-
cox2	5.14	+	-	7.7	+	-	12.84	-	-
ctsk	10.29	+	+	15.44	+	+	2.57	+	-
cyp2a6	0	-	-	0	-	-	0	-	-
dhfr	20.4	+	-	7.65	+	-	5.1	+	-

egfr	10.3	+	-	10.3	+	-	7.72	+	-
ephb4	25.29	+	+	25.29	+	+	17.7	-	-
er-beta	14.29	+	-	7.15	+	-	7.15	-	-
erbb2	29.6	+	+	29.6	+	+	22.2	-	-
fgfr1	27.02	+	+	27.02	+	+	14.74	+	-
fkbp1a	27	+	-	18.9	+	-	2.7	+	-
fxa	30.59	+	+	25.49	+	+	17.84	+	+
gba	5.15	+	+	0	+	+	2.57	-	-
gr	5.1	+	+	2.55	+	+	2.55	+	+
gsk3b	17.59	+	+	17.59	+	+	12.56	+	+
hdac2	10.12	+	+	7.59	+	+	2.53	+	-
hdac8	2.57	+	+	0	+	+	0	+	-
hiv1pr	30.13	+	+	30.13	+	+	24.65	+	+
hiv1rt	0	+	-	5.32	+	-	0	+	-
hmgr	23.19	+	-	28.35	+	-	30.92	+	-
hsp90	30.35	+	+	12.65	+	+	7.59	+	-
igf1r	2.48	+	+	4.97	+	+	2.48	-	-
inha	5.1	-	-	5.1	-	-	0	-	-
itk	7.61	+	+	7.61	+	+	12.69	+	-
jak3	29.93	+	+	19.95	+	+	9.97	+	-
jnk1	13.13	+	+	13.13	+	+	7.88	-	-
jnk2	17.51	+	+	17.51	+	+	10.01	+	+
jnk3	15.5	+	+	20.67	+	+	12.92	+	-
kif11	22.67	+	-	22.67	+	-	12.59	+	-
lck	20.07	+	+	20.07	+	+	15.05	+	-
mdm2	7.49	+	+	0	+	+	12.48	+	-
mk2	13.11	+	+	13.11	+	+	5.24	-	-
mmp2	2.55	-	-	0	-	-	2.56	-	-
na	30.98	+	+	30.98	+	+	30.98	-	-
p38-alpha	30.55	+	+	30.55	+	+	17.82	+	-
parp-1	27.61	+	+	25.1	+	+	7.53	+	+
pde4b	12.83	+	+	2.57	+	+	5.13	+	-
pde5	8.07	+	+	8.07	+	+	10.75	+	-
pdk1	31.74	+	+	31.74	+	+	7.93	+	-
pi3kg	20.68	+	+	18.1	+	+	20.68	-	-
pim-1	20.15	+	+	15.11	+	+	7.56	+	-
pim-2	18.07	+	-	7.74	+	-	7.74	-	-
pnp	30.83	+	+	30.83	+	+	10.28	+	+
ppara	8.17	+	-	5.44	+	-	0	-	-

pparg	12.95	+	+	12.95	+	+	0	+	+
pr	0	+	+	0	+	+	5.88	+	+
prkcq	26.32	+	+	26.32	+	+	26.32	+	+
pygl-in	25.67	+	-	25.67	+	-	2.57	-	-
pygl-out	10.3	+	-	7.73	+	-	28.33	-	-
qpct	18.05	+	+	7.74	+	+	7.74	+	+
rock-1	15.09	+	+	15.09	+	+	12.57	-	-
rxr	23.33	+	-	23.33	+	-	15.55	+	-
sars-hcov	2.57	+	+	2.57	+	+	0	+	+
sirt2	2.54	+	-	2.54	+	-	0	-	-
src	15.53	+	-	15.53	+	-	2.59	+	-
thrombin	29.21	+	-	29.21	+	-	15.93	+	-
tie2	14.99	+	+	14.99	+	+	5	-	-
tk	26.37	-	-	13.18	-	-	13.18	-	-
tp	0	+	+	0	+	+	3.28	+	+
tpa	30.55	+	+	30.55	+	+	2.55	+	-
ts	0	+	+	2.54	+	+	0	+	+
upa	29.9	+	+	29.9	+	+	9.97	+	+
vegfr1	16.33	+	+	13.61	+	+	5.44	-	-
vegfr2	23.46	+	+	26.07	+	+	7.82	+	-

Table S3. EF1% ranking performance of KarmaDock, RTMScore and CNNscore of gnina on pre-docked DEKOIS2 data set
